# An adaptive detection method for fetal chromosomal aneuploidy using cell-free DNA from 447 Korean women

**DOI:** 10.1186/s12920-016-0222-5

**Published:** 2016-10-03

**Authors:** Sunshin Kim, HeeJung Jung, Sung Hee Han, SeungJae Lee, JeongSub Kwon, Min Gyun Kim, Hyungsik Chu, Kyudong Han, Hwanjong Kwak, Sunghoon Park, Hee Jae Joo, Minae An, Jungsu Ha, Kyusang Lee, Byung Chul Kim, Hailing Zheng, Xinqiang Zhu, Hongliang Chen, Jong Bhak

**Affiliations:** 1 GenomeCare, Suwon, Republic of Korea; 2 Mirae & Heemang OB/GYN Clinic, Seoul, Republic of Korea; 3 Seoul Clinical Laboratories (SCL), Yongin, Republic of Korea; 4 Namujungwon Maternity Hospital, Yangju, Republic of Korea; 5 GN Maternity Hospital, Pyeongtak, Republic of Korea; 6 Department of Nanobiomedical Science, BK21 PLUS NBM Global Research Center for, Regenerative Medicine, Dankook University, Cheonan, Republic of Korea; 7 TheragenEtex, Suwon, Republic of Korea; 8 The Genomics Institute (TGI), BioMedical Engineering, UNIST, Ulsan, 687-798 Republic of Korea; 9 Xiamen Vangenes BioTech, Xiamen, Fujian China; 10 Geromics, Ulsan, 687-798 Republic of Korea; 11 Genome Research Foundation, Osong, Chungbuk Republic of Korea

**Keywords:** Non-invasive prenatal testing, Adaptive detection algorithm, Sequencing, Circulating fetal DNA, Trisomy, Genome

## Abstract

**Background:**

Noninvasive prenatal testing (NIPT) using massively parallel sequencing of cell-free DNA (cfDNA) is increasingly being used to predict fetal chromosomal abnormalities. However, concerns over erroneous predictions which occur while performing NIPT still exist in pregnant women at high risk for fetal aneuploidy. We performed the largest-scale clinical NIPT study in Korea to date to assess the risk of false negatives and false positives using next-generation sequencing.

**Methods:**

A total of 447 pregnant women at high risk for fetal aneuploidy were enrolled at 12 hospitals in Korea. They underwent definitive diagnoses by full karyotyping by blind analysis and received aneuploidy screening at 11–22 weeks of gestation. Three steps were employed for cfDNA analyses. First, cfDNA was sequenced. Second, the effect of GC bias was corrected using normalization of samples as well as LOESS and linear regressions. Finally, statistical analysis was performed after selecting a set of reference samples optimally adapted to a test sample from the whole reference samples. We evaluated our approach by performing cfDNA testing to assess the risk of trisomies 13, 18, and 21 using the sets of extracted reference samples.

**Results:**

The adaptive selection algorithm presented here was used to choose a more optimized reference sample, which was evaluated by the coefficient of variation (CV), demonstrated a lower CV and higher sensitivity than standard approaches. Our adaptive approach also showed that fetal aneuploidies could be detected correctly by clearly splitting the z scores obtained for positive and negative samples.

**Conclusions:**

We show that our adaptive reference selection algorithm for optimizing trisomy detection showed improved reliability and will further support practitioners in reducing both false negative and positive results.

**Electronic supplementary material:**

The online version of this article (doi:10.1186/s12920-016-0222-5) contains supplementary material, which is available to authorized users.

## Background

In 1997 Lo et al*.* reported that Y-chromosome derived, male, cell-free fetal DNA exists in maternal female blood plasma and serum similar to tumor DNA using a polymerase chain method [1]. Since then, molecular screening of cell-free DNA (cfDNA) for detecting fetal aneuploidy has generated much interest because aneuploidy and other chromosome aberrations are fairly common (nine out of 1,000 live births) [2]. As a result, the discovery has inspired the development of many detection methods [3]. However, the main obstacle in the development of fast and low-cost diagnostic assays remains the low fraction (<4 %) of cell-free, fetal DNA in mothers [4]. Especially when cell-free fetal DNA is less than 3.5 %, the number of unique DNA fragments increases exponentially to retain the required aneuploidy detection power [5]. In addition, detecting fetal aneuploidy at an early diagnostic stage is still difficult because the fraction of original fetal DNA is proportional to gestational age [6]. Earlier detection could facilitate further diagnoses and actions. In twin pregnancies, it is more difficult to detect fetal aneuploidy because the fetal fraction (FF) of an affected fetus may be far lower than 4 % [7]. FF could be reduced by 50 % owing to the proportion of a second normal fetus.

A high risk of fetal aneuploidy has been identified by the first or second trimester screening, including maternal age, ultrasound and maternal serum markers [8]. Women at high risk are subjected to invasive sampling of fetal materials by amniocentesis for gestational age at week 15 and by chorionic villus sampling for gestational age at week 12 [9, 10]. However, these tests carry the risk of iatrogenic pregnancy loss [11]. CfDNA screening, on the other hand, offers two, major, clinical benefits compared to invasive prenatal diagnoses: no risk of pregnancy loss and earlier detection. CfDNA screening does have several limitations, such as requirements for further invasive tests to confirm positive outcomes in the case of discordant results that might arise from placental or maternal cell mosaicism [12–14], the average size of cfDNA being only around 150 base pairs (bp) [15] and short half-life [16]. Even with these shortcomings, sequencing-based, cfDNA screening using statistically improved counting methods has risen in popularity among pregnant women [17–19].

Since cfDNA screening for fetal aneuploidy was introduced, reducing GC bias to detect aneuploidy with higher sensitivities by reducing the coefficient of variation (CV) has become a key issue. Fan et al. [17], for example, detected fetal aneuploidy initially by counting the number of unique reads within each sliding window, enabling clear separation of fetal trisomy outliers. They successfully detected nine cases of trisomy 21 (T21), two cases of T18, and one case of T13 in a cohort of 18 pregnancies by measuring sequence tag density relative to the corresponding value of the genome DNA control to remove GC bias representing the higher GC content. Meanwhile, Chiu et al. suggested a method of detecting fetal aneuploidy involving counting the unique reads mapped to each chromosome and calculating z-scores with the percentage of all the unique reads of each chromosome for a sample [18]. They correctly detected 14 T21 fetuses and 14 euploid fetuses with z score > 3 without considering GC bias; however, the higher GC content for chromosome X produced a smaller z score. They also performed a large-scale validity study using a previously established method that employs next-generation sequencing to detect fetal trisomy 21 in high-risk pregnancies with high accuracy. They detected 86 T21 fetuses with 100 % sensitivity and 97.9 % specificity among 753 pregnancies [20]. Jiang et al. improved cfDNA screening by employing GC-correlation to minimize GC-bias and estimate the fraction of cell-free fetal DNA as a key index to detect autosomal and sex chromosome aneuploidies with high accuracy [5]. In a total of 903 pregnancies, they detected autosomal aneuploidies with 100 % sensitivity and 99.9 % specificity, and sex chromosome aneuploidies with 85.7 % sensitivity and 99.9 % specificity by employing GC-correlation and data normalization. Recently, Liao et al. [21], reported a methodology used to systematically detect both autosomal and sex chromosomal aneuploidies with high accuracy. They employed an integrated method for GC correction, which includes LOESS regression, normalization and linear regression to reduce the effect of GC bias in a total of 515.

Despite these advances, the risks of false negatives and false positives still exist. In particular, cfDNA screening at low or high risk for fetal trisomy generates more false negative and false positive results [21]. In this study, we designed a new algorithm based on selecting reference samples adaptively according to the shared ranges of GC content and DNA reads fraction of a test sample (The GC-related terminologies used here were defined in Table 1).

**Table 1:** GC-related terminologies

Terminologies	Definition
GC content	The percentage of guanine and cytosine nitrogenous bases
GC content of a sample	The GC content of all unique reads of each chromosome of a sample, which are mapped to the corresponding chromosome of the reference genome
GC range	The range of GC content of a sample
Unit value of GC content	A unit value used to increase or decrease the range of GC content of each chromosome of a sample (default = 0.001)
The GC value of a test sample	The GC content of all unique reads of each chromosome of a test sample
Reads fraction of a sample	The percentage of all unique reads of each chromosome of a sample, which are mapped to each corresponding chromosome of reference genome
Unit value of reads fraction	A unit value used to increase or decrease the range of reads fraction of each chromosome of a sample (default = 0.00005)
The RF value of a test sample	The reads fraction value of all unique reads of each chromosome of a test sample, which was determined by fitting predicted fraction of reads calculated as $R{f}_{i^{\prime }{j}^{\prime}}^{\hbox{'}} = \upalpha +\upbeta \times G{C}_{i^{\prime }{j}^{\prime }}$ from all reference samples

## Methods

### Study participants and testing methods

From December 2014 through April 2015, 447 women at high risk for fetal aneuploidy were enrolled into this study from 12 hospitals (Mirae & Heemang, Namujungwon, and GN and others in Korea). The characteristics of the pregnant women are outlined in Table 2. The mean maternal age was 35, and ranged from 25 to 42 years. The mean gestational age was 15 weeks, and ranged from 11 to 22. Of these women, 29 were carrying twins, and their features are outlined in Table 3. All 447 women endured invasive prenatal diagnostic testing (amniocentesis) for fetal karyotyping, the results of which were obtained by blind analysis. The institutional review board at each participating hospital approved this study. Written informed consent was obtained from all participants.

**Table 2:** Demographic characteristics in 447 pregnancies. Demographic characteristics of 447 pregnant women in 12 hospitals in Korea

Characteristic	Value
No. of patients	447
Maternal age - year	
Mean	35
Range	20 ~ 46
Gestational age - week	
Mean	15
Median	16
Range	11 ~ 22
Pregnancy trimester - no. (%)	
First: 1–13 week gestation	137 (30.6)
Second: 14–26 week gestation	310 (69.4)
Male fetus - no. (%)	249 (52.5)
Female fetus - no. (%)	225 (47.5)

**Table 3:** Demographic characteristics in 29 twin pregnancies. Demographic characteristics of 29 twin pregnancies from 12 hospitals in Korea

Characteristic	Value
No. of patients with twins	29
Maternal age - year	
Mean	35
Range	22 ~ 43
Gestational age - week	
Mean	14
Median	13
Range	11 ~ 21
Pregnancy trimester - no. (%)	
First: 1–13 week gestation	16 (55.2)
Second: 14–26 week gestation	13 (44.8)
Male fetus - no. (%)	26 (48.1)
Female fetus - no. (%)	28 (51.9)

Two patients with unknown fetal sex were excluded

All women underwent standard prenatal aneuploidy screening using accredited clinical laboratories. First-trimester screening includes the measurement of serum pregnancy-associated plasma protein A, total or free beta subunit of human chorionic gonadotropin (hCG), and nuchal translucency. Second-trimester screening comprises measuring maternal serum alpha-fetoprotein, hCG, unconjugated estriol and inhibin A.

### CfDNA preparation and maternal plasma DNA sequencing

About 10 mL of peripheral blood was collected from each participant in a BCT™ tube (Streck, Omaha, NE, USA). The blood sample was centrifuged at 1,200 × g for 15 min at 4 °C. The plasma portion of blood was transferred to microcentrifuge tubes and centrifuged again at 16,000 × g for 10 min at 4 °C. CfDNA was extracted from 1 mL of plasma using a QIAamp Circulating Nucleic Acid Kit (Qiagen, Netherland). The end-repair of the plasma cfDNA was carried out using T4 DNA polymerase, Klenow DNA polymerase, and T4 polymerase kinase. DNA libraries for the Ion Proton sequencing systems were constructed according to the protocol provided by the manufacturer (Life Technologies, SD, USA). Proton PI Chip Kit version 2.0 was used to yield an average 0.3× sequencing coverage depth per nucleotide.

### Data analysis

We used three processing steps for our comparative cfDNA analyses. First, cfDNA was sequenced massively using the Ion Proton system. All raw reads obtained from the Ion Torrent Suite Software (Life Technologies) were trimmed from the 3′ end using a sequencing quality threshold value of 20 (Q score) and filtered by a read length threshold of 50 bp. The remaining reads were aligned to the human genomic reference sequences (hg19) using BWA [22]. Duplicate DNA reads were filtered out by the Picard program (http://broadinstitute.github.io/picard/). Second, the effect of GC bias was reduced using LOESS regression [23], normalization of samples [5] and linear regression [24]. Each chromosome was divided into bins of 20 kb. After LOESS correction [24], given the corrected unique reads (*RC*_*ij*_) on chromosome *j* of sample *i*, the fraction of reads ($R{f}_{i{j}^{\hbox{'}}}$) was calculated as follows: $R{f}_{i{j}^{\hbox{'}}}=R{C}_{ij}/{\displaystyle \sum_{j=1}^{22}}R{C}_{ij}$. The normalization of samples was calculated as follows: $R{f}_{i^{\prime }{j}^{\prime }}=R{f}_{i{j}^{\hbox{'}}}/{\displaystyle \sum_{i=1}^N}R{f}_{i{j}^{\hbox{'}}}$. The final step was to perform a statistical analysis after selecting reference samples adapted to a test sample from all the reference samples [24]. The full linear regression model was established based on equation, $R{f}_{i^{\prime }{j}^{\prime }}=\alpha + \beta \times G{C}_{i^{\prime }{j}^{\prime }}+\mathrm{e}$ where $G{C}_{i^{\prime }{j}^{\prime }}$ was the GC content of chromosome *j’* of sample *i’*, *β* was the coefficient factor between the fraction of reads and GC content, and e was the error term. Fitting of the predicted fraction of reads was calculated as $R{f}_{i^{\prime }{j}^{\prime}}^{\hbox{'}} = \upalpha +\upbeta \times G{C}_{i^{\prime }{j}^{\prime }}$. The residual obtained by the equation $\mathrm{R} = R{f}_{i^{\prime }{j}^{\prime }}-R{f}_{i^{\prime }{j}^{\prime}}^{\hbox{'}}$ was fitted to a normal distribution and was used to derive a z score for fetal aneuploidies [24].

Optimally adaptive reference samples were extracted from all reference samples belonging to a shared range of the GC content and the reads fraction of a test sample as shown in Additional file 1: Figure S1. The GC content range in this study was set from −0.001 to +0.001 as a stepping unit value when setting the GC content of a test sample as the median. The reads fraction range was set from −0.00005 to +0.00005 as a stepping unit value when setting the reads fraction of a test sample as the median, which was determined by the fitting predicted fraction of reads calculated as $R{f}_{i^{\prime }{j}^{\prime}}^{\hbox{'}} = \upalpha +\upbeta \times G{C}_{i^{\prime }{j}^{\prime }}$ from all reference samples. The representative sample in each test group of samples was selected and used to generate a set of reference samples increasing by 0.001 in GC content and by 0.00005 in reads fraction. By adjusting the unit value of GC content or reads fraction, the resolution of sets of reference samples could be changed. That is, a smaller unit could make more conservative ranges of sets, while a larger unit could make less conservative ranges of sets. In this study, changes of 0.001 in GC content and 0.00005 in reads fraction were set as the default stepping unit values.

We reasoned that a suboptimal threshold could result from a suboptimal reference sample collection that is not adapted statistically optimally to the test sample. Therefore, we tried to collect a set of more optimized, or “adaptive”, reference samples. First, the positive samples in 0.01 intervals of GC content value were categorized into four groups of 0.41, 0.42, 0.43, and 0.44 GC content regions. This was to efficiently collect adaptive reference samples before extending a shared range of the GC content and the reads fraction of a test sample using the unit value of GC content or reads fraction. Thus, the two positive test samples in the 0.41 GC content region, the five positive test samples in the 0.42 region, the two positive test samples in the 0.43 region, and the four positive test samples in the 0.44 region were clustered according to the GC regions, respectively. Second, if a sample size was >2, the median G + C content in each group was chosen as the representative test sample. If there were only 2 samples in a group, a representative was arbitrarily chosen. Third, the representative sample was used to generate a set of reference samples by increasing the GC content by 0.001 and the reads fraction by 0.00005. This sets the common region shared with the GC content of the representative sample ± 0.001 and the reads fraction of the representative sample ± 0.00005, to generate a set of reference samples from the shared region. We repeated this to extend the reads fraction of the representative sample by ±0.0001 (increasing an absolute unit value of reads fraction) until the reads fraction reached the preset threshold (±0.001). We repeated this after extending the GC content of the representative sample by ±0.002 (increasing the absolute unit value of GC content) until the GC content reached the preset threshold (±0.02). Finally, sets of optimized reference samples were selected by checking the CVs, which were used to evaluate the quality of the set of reference samples.

A z score > 3 indicated the fraction of chromosome reads greater than that of the 99.9th percentile of the set of the reference samples for a one-tailed distribution [18]. We evaluated our method by performing cfDNA testing to assess the risk of trisomies 21, 18, and 13.

## Results

From 447 plasma samples with existing karyotyping diagnoses, we showed that the adaptive selection strategy of reference samples produced a more reliable and robust result than the previous approach of using all reference samples. There were 13 fetuses with T21 (including three twin samples), one fetus with T18 in a twin pregnancy, one fetus with T13, and two fetuses with XXY. Seventeen samples with aneuploidy, 29 samples with twins, and five samples recognized as outliers were excluded from 447 samples to produce more reliable reference samples. Thus, we compared the adaptive selection method with the non-adaptive selection method using 396 reference samples. An average of approximately 7.4 ± 2.1 million raw reads were obtained per sample. When sequence reads mapped to only one genome location in the reference human genome, they were termed unique reads. Approximately 44.6 %, or 3.3 million unique reads, of the total raw reads were retained. The distribution of GC contents of these 396 samples ranged from 40 % to 51 %. The CV to evaluate the performance between the traditional and new methods were used.

**Fig. 1:**
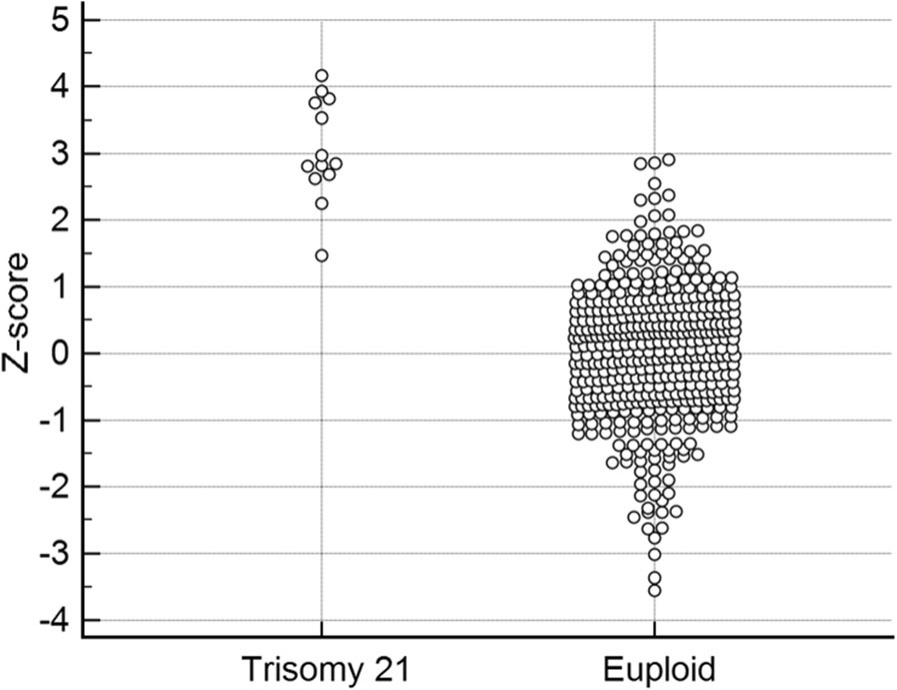
Z scores obtained using the previous method. Z scores obtained for each sample along with the ambiguous threshold obtained using the previous method for chromosome 21 using reference samples (*n* = 396)

**Fig. 2:**
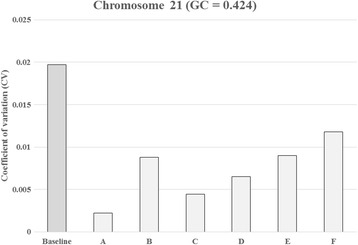
Coefficient of variation (CV) for chromosome 21 with and without adaptive sample selection using the representative sample with a GC = 0.424. The baseline bar represents the coefficient of variation used to measure the genomic representation of chromosome 21 among reference samples (*n* = 396) without adaptive sample selection. Additional bars represent the CV with adaptive sample selection. The bar marked A represents the coefficient of variation used to measure the genomic representation of chromosome 21 among selected reference samples (*n* = 37). The B (*n* = 210), C (*n* = 120), D (*n* = 166), E (*n* = 226), and F (*n* = 278) also represent the CV with various numbers of reference samples

**Fig. 3:**
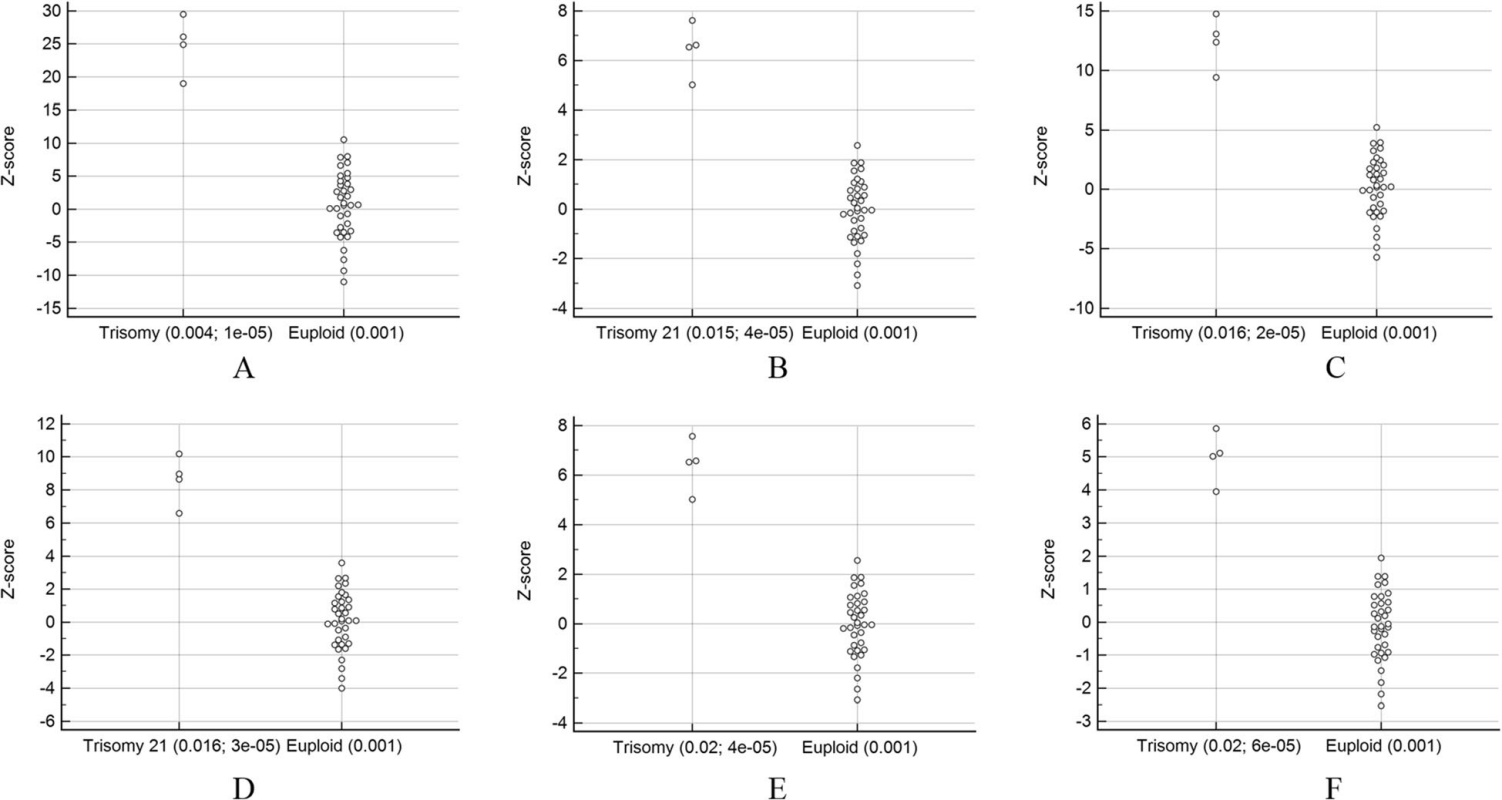
Z scores obtained in the **a**, **b**, **c**, **d**, **e**, and **f** sets of adaptive reference samples generated using the adaptive method. Z scores obtained for each sample along with the unambiguous thresholds using the adaptively selected samples represented in Fig. 2 are shown. The study included five T21 samples containing GC contents of the 0.42 region. The first (a representative test sample) was used to select adaptive samples according to a GC range and a reads fraction range of the representative sample. For example, 0.004 and 1e-05 represent a GC range and a reads fraction range of the representative sample, respectively, in the A set of adaptive reference samples. The others were used to test positive results using the adaptive reference samples. The euploid samples within 0.424 ± 0.001 were selected to test negative results

As shown in Additional file 1: Figure S2, GC correction played an important role in reducing the CV. Bars represent the CV for chromosomes 13, 18, and 21 with and without LOESS-based GC correction among reference samples (*n* = 396). However, despite the GC correction of the samples, Fig. 1 shows that the threshold is still suboptimal in separating positive and negative results using a traditional method for chromosome 21 perhaps due to suboptimal reference samples. Figure 2 shows the CV for chromosome 21 with and without adaptive sample selection using a representative sample with a GC = 0.424. Every CV for the adaptive approach was lower than those for the baseline approach. Therefore, the adaptive approach provided higher sensitivity for T21. In fact, Fig. 3 shows the clear thresholds obtained using the six sets of adaptively selected samples. One, a representative test sample, of five T21 samples containing GC contents in the 0.42 region was used to select adaptive samples according to a GC range and a reads fraction range of the representative sample. The remaining four T21 samples were used to evaluate positive results using the six sets. We also selected six sets of the euploid samples within 0.424 ± 0.001 to evaluate negative results.

**Fig. 4:**
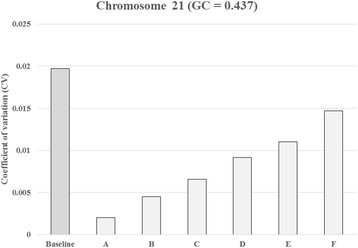
Coefficient of variation (CV) for chromosome 21 with and without adaptive sample selection using the representative sample with a GC = 0.437. The baseline bar represents the coefficient of variation used to measure the genomic representation of chromosome 21 among reference samples (*n* = 396) without adaptive sample selection. Additional bars represent the CV with adaptive sample selection. The bar marked A represents the coefficient of variation used to measure the genomic representation of chromosome 21 among selected reference samples (*n* = 31). The B (*n* = 90), C (*n* = 138), D (*n* = 189), E (*n* = 227), and F (*n* = 292) also represent the CV with increased numbers of reference samples

**Fig. 5:**
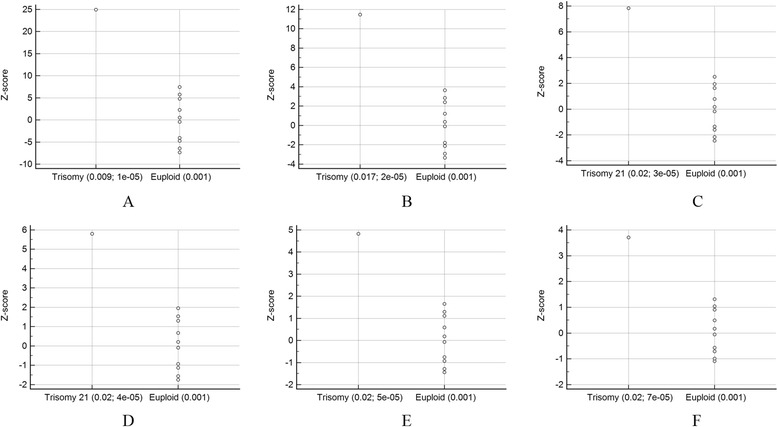
Z scores obtainedin the **a**, **b**, **c**, **d**, **e**, and **f** sets of adaptive reference samples generated with the adaptive method. Z scores obtained for each sample along with the unambiguous thresholds using the adaptively selected samples represented in Fig. 4 are shown. The study included two T21 samples containing GC contents of the 0.43 region. The first (a representative test sample) was used to select adaptive samples according to a GC range and a reads fraction range of the representative sample. For example, 0.009 and 1e-05 represent a GC range and a reads fraction range of the representative sample, respectively, in the A set of adaptive reference samples. The second was used to test the positive result using the adaptive samples. The euploid samples within 0.437 ± 0.001 were selected to test negative results

Figures 4 and 5 show similar results with adaptive sample selection using a representative sample with a GC = 0.437. In addition, Additional file 1: Figure S3.1 and Additional file 1: Figure S3.2, using a representative sample with a GC = 0.416, and Additional file 1: Figure S4.1 and Additional file 1: Figure S4.2, using a representative sample with a GC = 0.446, show similar results to our adaptive sample selection. As we had only one T18 and one T13 sample, we could not test these results using other T18 and T13 samples. However, we found that only one T18 or T13 reference could generate a good set of adaptively selected samples to clearly separate the z scores obtained for positive and negative samples (Additional file 1: Figure S5 and Additional file 1: Figure S6). A significant linear model was set up to analyze the relationship of the reads fractions and the GC contents of samples (Additional file 1: Figure S7).

Notably, we correctly detected three T21 and one T18 aneuploid samples in twin pregnancies. Currently, it requires an FF of at least 4 % to get reliable results for accurate cfDNA analyses [20, 25–30]. In our twin pregnancies, three positive T21 results were dizygotic twin pregnancies and one positive T13 result was a monozygotic twin pregnancy. Therefore, the three positive results could have been false negatives because the FF of the affected fetus could be below the 4 % threshold. Instead of determining FF in this study, we used the z score of aneuploid chromosomes, which shows a positive correlation with FF [24] as it is difficult to determine FF precisely. Notably, setting cutoff values of the z score as 2 for a negative result and 4 for a positive result showed that the specific results of this study satisfied the criteria (Fig. 3; f, Fig. 5; d, e).

## Discussion

We have noted that the number of unique reads is correlated statistically with the GC content [5]. Therefore, obtaining robust results for fetal aneuploidy detection suggests the hypothesis that the GC content of the sample under test belongs to the range of GC contents of the reference samples. The reason for this being that the key criteria for detecting fetal aneuploidy in a test sample is the fitting of the predicted value from the reference samples. Thus, the predictability of the state of the test sample depends on the statistical state of the reference samples. Therefore, we applied this concept to detect fetal aneuploidies by selecting the reference samples adaptively according to the GC content of a test sample.

We observed that in the process of selecting adaptive reference samples, the range of the reads fraction of a test sample is important to the collection of suitable reference samples. Therefore, we investigated the adaptive reference samples belonging to the shared region of a GC content value and a reads fraction value.

In earlier studies, cfDNA screening for fetal aneuploidy was performed successfully using smaller sized, reference samples [17, 18]. Fan et al. [17] successfully detected 12 fetal aneuploidies by counting the number of unique reads within each sliding window and separating the outliers of fetal trisomy clearly with six reference samples using the higher sample GC contents of (range, 42 % to 50 %). The GC distribution of these samples was very similar to the GC distribution of our 396 reference samples (GC range, 40 % to 51 %). We investigated why the previous method did not detect fetal aneuploidy correctly, although it used a comparatively large number (*n* = 396) of reference samples. Considering the distribution of reads fractions *vs*. GC contents of samples as shown in Additional file 1: Figure S7, we hypothesized that the reason was the unbalanced distribution of the reads fractions of samples according to the increasing GC content values, especially at higher GC contents. On the other hand, Liao et al. [24] detected aneuploidies with high accuracy in 515 pregnancies (GC range, 38 % to 42 %) with a comparatively balanced distribution of reads ratios *vs.* GC contents of samples. Nevertheless, their results also had borderline values of the z scores of chromosome 21 for positive and negative samples. This means that the quality of a set of reference samples is more important than the sample size. Until now, mainstream cfDNA screening has focused on the issue of reducing GC bias by increasing the sample size. This study suggested an adaptive selection approach to collect samples adaptively according to the GC content of a test sample.

Although this approach was practically feasible in our data for detecting chromosome 21 aneuploidy, an independent, larger sample size is required to confirm our results. A sufficiently large sample size is necessary to decide how many reference samples would be required to obtain sufficient evidence of the reliability and validity of our results. In addition, although only one T18 or T13 test sample could generate a good set of adaptively selected samples (Additional file 1: Figure S5 and Additional file 1: Figure S6), we need to confirm our results by detecting T18 or T13 in independent positive samples, using the set of selected reference samples.

## Conclusions

Using 447 samples, we developed a new adaptive method of selecting reference samples according to the combined values of the GC content and the reads fraction of a test sample. The approach was compared with the previous method using all reference samples in order to detect fetal aneuploidy and demonstrated to be reliable and robust.

## Additional file

Additional file 1:
**Figure S1** showed optimally adaptive reference samples extracted from all reference samples. **Figure S2** showed that GC correction played an important role in reducing the CV. **Figures S3.1, S3.2, S4.1, S4.2, S5** and **S6** represented similar results to our adaptive sample selection. **Figure S7** represented the relationship of the reads fractions and the GC contents of samples. (DOCX 2063 kb)

## Abbreviations

bp: Base pairs
cfDNA: Cell-free DNA
CV: Coefficient of variation
hCG: Human chorionic gonadotropin
NIPT: Non-invasive prenatal testing
SD: Standard deviation
