# Supplementary material for: An adaptive detection method for fetal chromosomal aneuploidy using cell-free DNA from 447 Korean women
Source: BMC Med Genomics. 2016 Oct 3;9:61. doi: 10.1186/s12920-016-0222-5 (PMC5048604; doi:10.1186/s12920-016-0222-5)
Supplement: Additional file 1: — Figure S1 showed optimally adaptive reference samples extracted from all reference samples. Figure S2 showed that GC correction played an important role in reducing the CV. Figures S3.1, S3.2, S4.1, S4.2, S5 and S6 represented similar results to our adaptive sample selection. Figure S7 represented the relationship of the reads fractions and the GC contents of samples. (DOCX 2063 kb) [file 12920_2016_222_MOESM1_ESM.docx]

Supporting Information

An adaptive detection method for fetal chromosomal aneuploidy using cell-free DNA from 447 Korean women

Sunshin Kim, HeeJung Jung, Sung Hee Han, SeungJae Lee, JeongSub Kwon, Min Gyun Kim, Hyungsik Chu, Kyudong Han, Hwanjong Kwak, Sunghoon Park, Hee Jae Joo, Minae An, Jungsu Ha, Kyusang Lee, Byung Chul Kim, Hailing Zheng, Xinqiang Zhu, Hongliang Chen, and Jong Bhak

**Figure S1 – Adaptive sample selection from whole reference samples**

The GC content range in this study was set from –0.001 to +0.001 as a unit value when setting the GC content of a test sample as the median. The reads fraction range was also set from –0.00005 to +0.00005 as a unit value when setting the reads fraction of a test sample as the median, which was determined by fitting the predicted fraction of reads calculated as ${Rf}_{i^{'}j^{'}}^{'}= \alpha+\beta\times{GC}_{i^{'}j^{'}}$ from all reference samples.


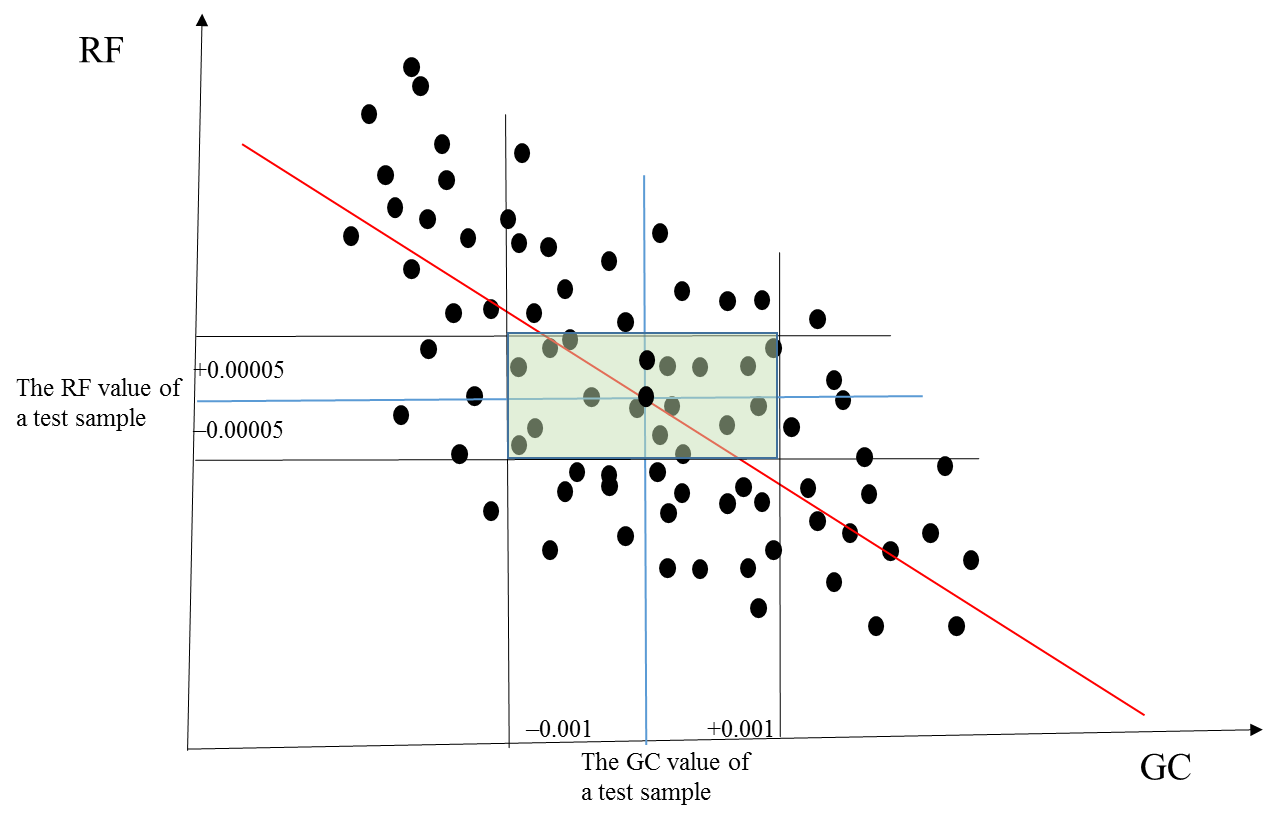


**Figure S2 – Coefficients of variation (CVs) for chromosomes 13, 18, and 21 with and without LOESS-based GC correction**

Both bars represent the coefficients of variation used to measure the genomic representation of chromosomes 13, 18, and 21 with and without LOESS-based GC correction among reference samples (*n* = 396).

**
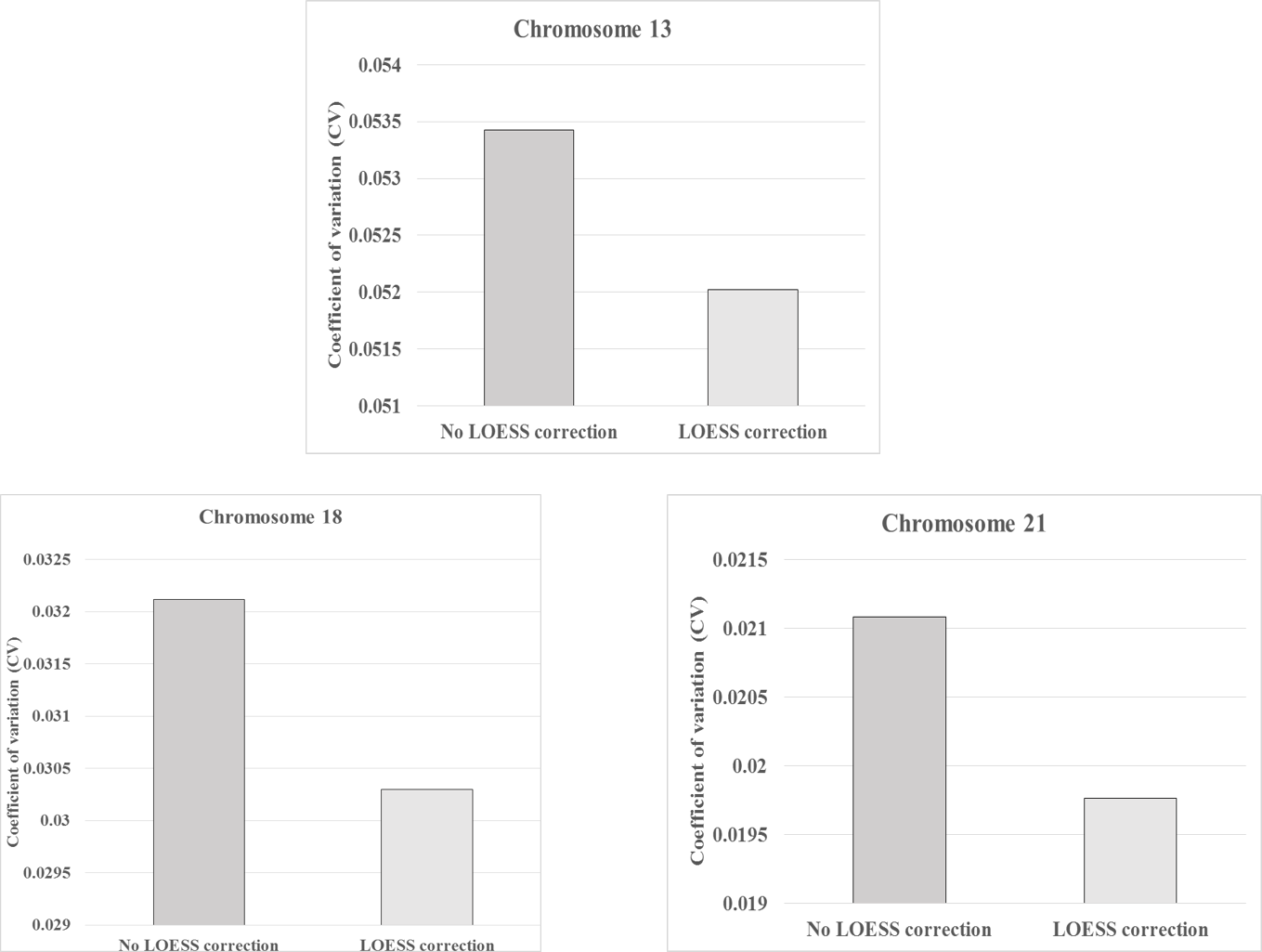
**

**Figure S3.1 – Coefficient of variation (CV) for chromosome 21 with and without adaptive sample selection using a representative sample with GC = 0.416**

The baseline bar represents the coefficient of variation used to measure the genomic representation of chromosome 21 among reference samples (*n* = 396) without adaptive sample selection. Additional bars represent the CV with adaptive sample selection. The bar marked A represents the coefficient of variation used to measure the genomic representation of chromosome 21 among selected reference samples (*n* = 27). The B (*n* = 110), C (*n* = 157), D (*n* = 195), E (*n* = 246), and F (*n* = 276) also represent the CV with increased numbers of reference samples.


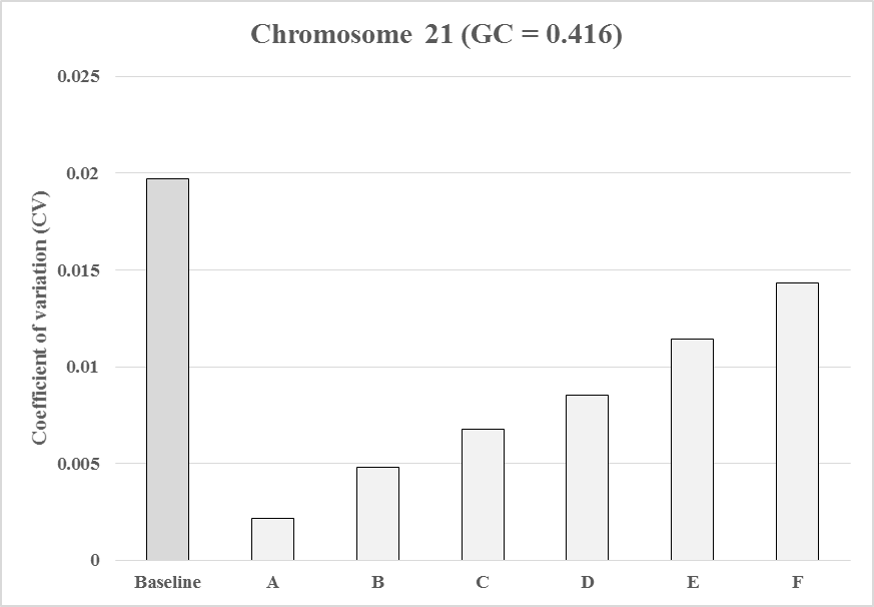


**Figure S3.2 – Z scores using the adaptive method**

Z scores obtained for each sample along with the unambiguous thresholds using the adaptively selected samples represented in Figure S3.1 are shown. The study included two T21 samples containing GC contents of the 0.41 region. The first (a representative test sample) was used to select adaptive samples according to a GC range and a reads fraction range of the representative sample. For example, 0.009 and 1e-05 represent the GC range and reads fraction range of the representative sample, respectively, in the A set of adaptive reference samples. The second was used to test the positive result using the adaptive samples. The euploid samples within the range of 0.416 ± 0.001 were selected to test negative results.

**
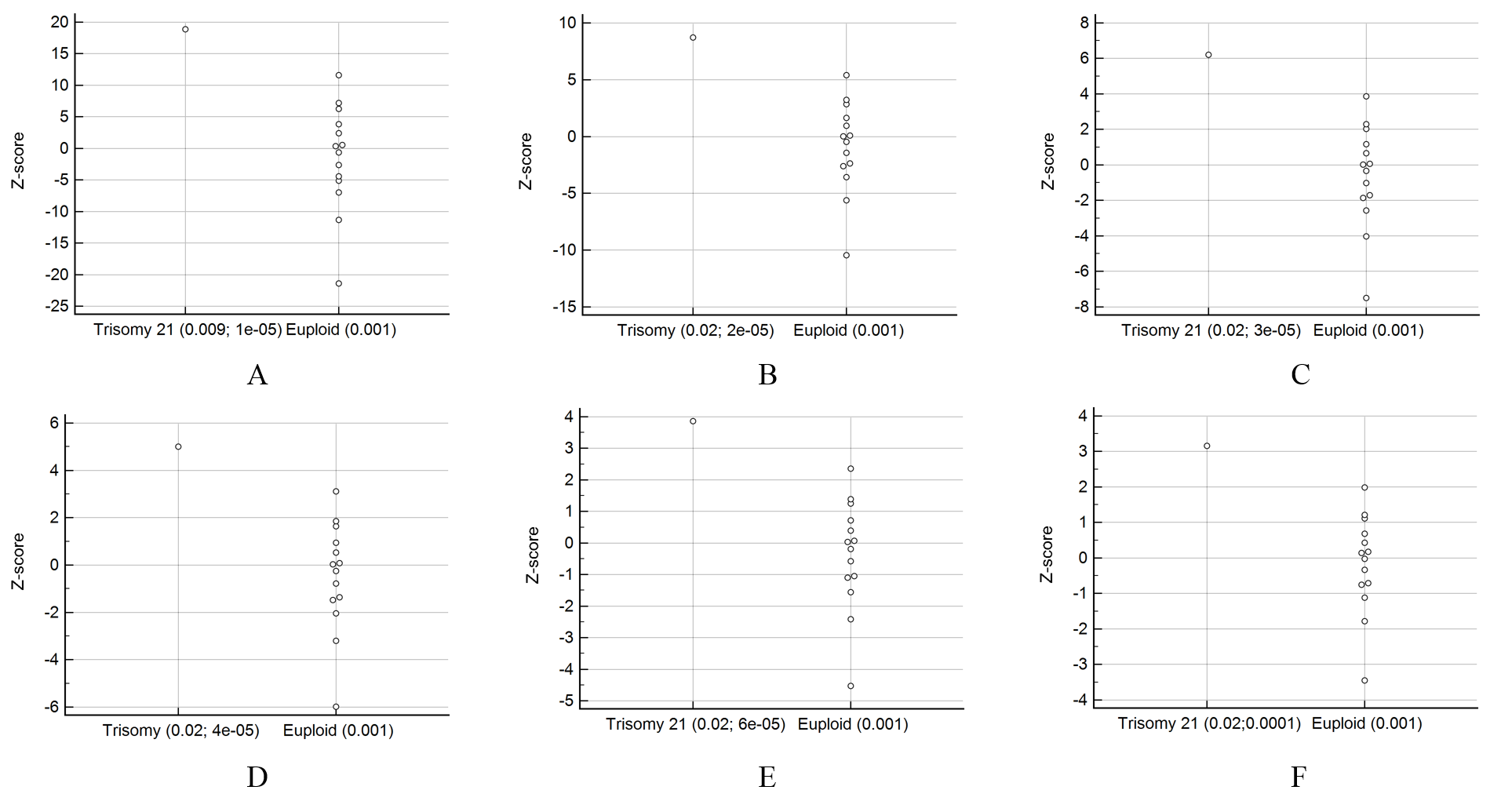
**

**Figure S4.1 – Coefficient of variation (CV) for chromosome 21 with and without adaptive sample selection using the representative sample with GC = 0.446**

The baseline bar represents the coefficient of variation used to measure the genomic representation of chromosome 21 among reference samples (*n* = 396) without adaptive sample selection. Additional bars represent the CV with adaptive sample selection. The bar marked A shows the coefficient of variation used to measure the genomic representation of chromosome 21 among selected reference samples (*n* = 38). The B (*n* = 127), C (*n* = 93), and D (*n* = 181) also represent the CV with various numbers of reference samples.


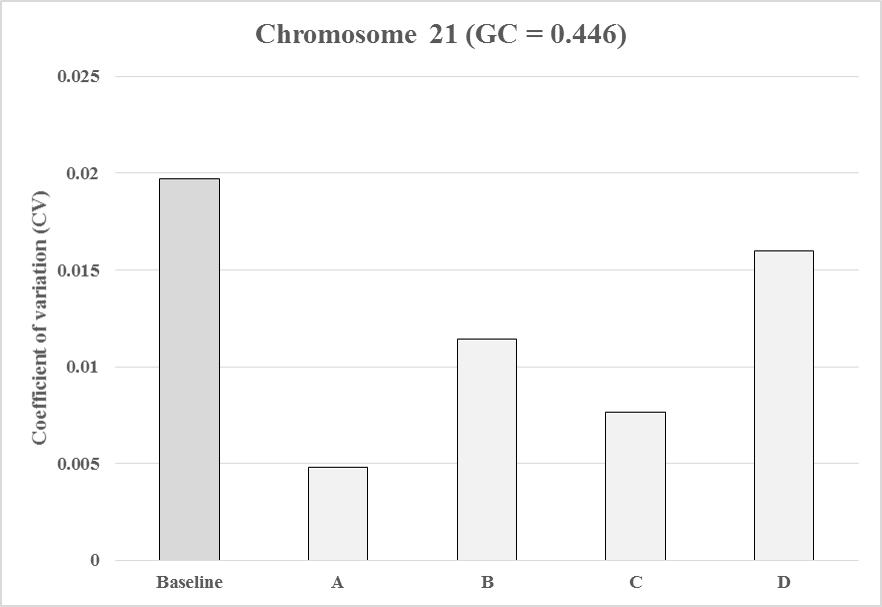


**Figure S4.2 – Z scores using the adaptive method**

Z scores obtained for each sample along with the unambiguous thresholds using the adaptively selected samples represented in Figure S4.1 are shown. The study included four T21 samples containing GC contents in the 0.44 region. The first (a representative test sample) was used to select adaptive samples according to a GC range and a reads faction range of the representative sample. For example, 0.011 and 2e-05 represent a GC range and a reads fraction range of the representative sample, respectively, in the A set of adaptive reference samples. The others were used to test positive results using the adaptive samples. The euploid samples within 0.446 ± 0.001 were selected to test negative results.


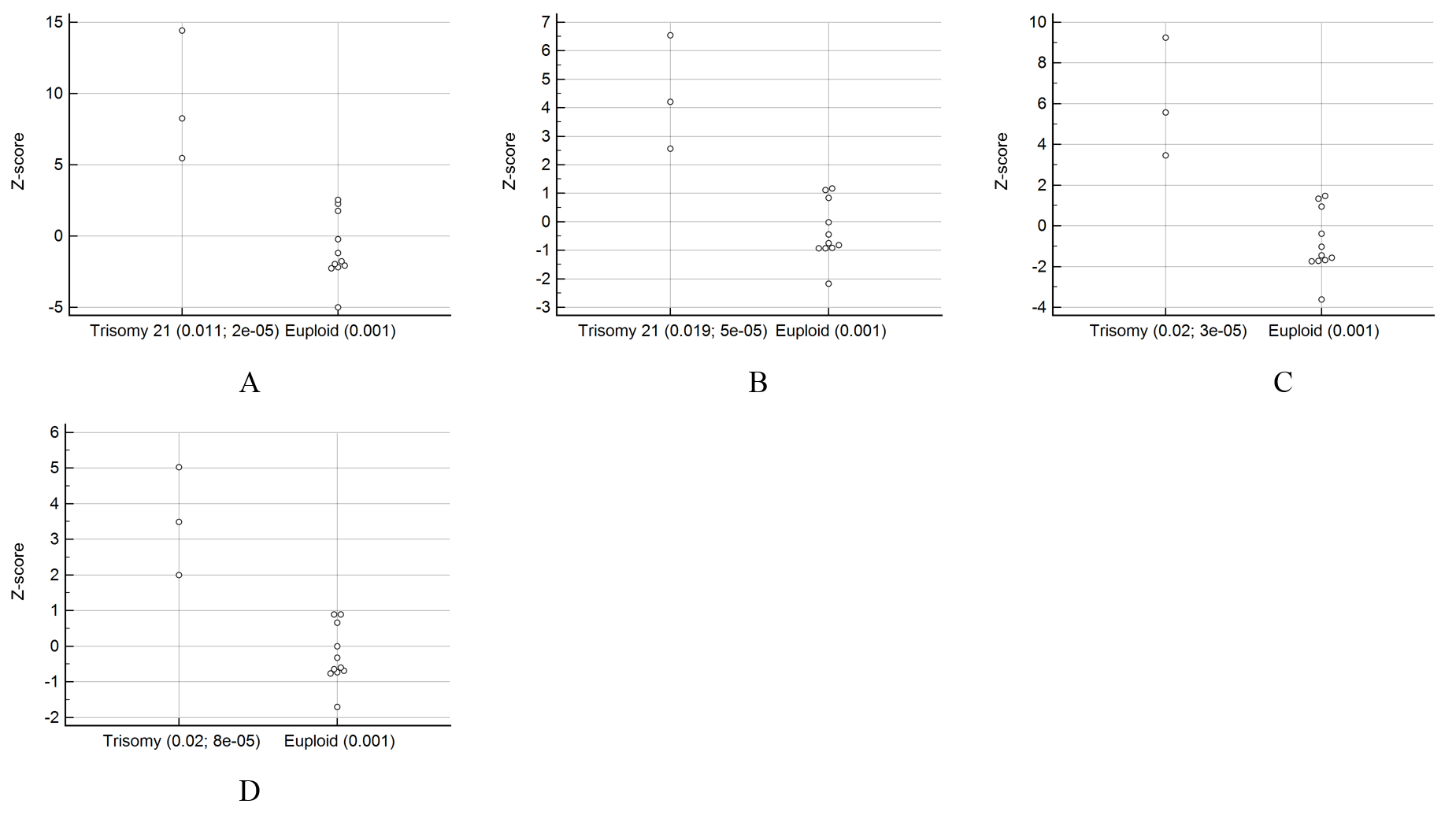


**Figure S5 – Coefficient of variation (CV) and z scores for chromosome 18 with and without the adaptive method using the only sample with GC = 0.45**

The baseline bar represents the coefficient of variation used to measure the genomic representation of chromosome 18 among reference samples (*n* = 396) without using adaptive sample selection. The additional bar represents the CV with adaptive sample selection. The bar marked A represents the coefficient of variation used to measure the genomic representation of chromosome 18 among selected reference samples (*n* = 8). Z scores obtained for each sample along with the ambiguous threshold using the previous method for chromosome 18 and the unambiguous threshold using the adaptive method for chromosome 18 are shown. We had only one T18 sample and could not test the results using the other T18 samples. However, we found that only the T18 sample could generate a good set of adaptively selected samples (*n* = 8), which represented 0.014 and 2e-05 for a GC range and a reads fraction range of the test sample, respectively, in the set of adaptive reference samples. The euploid samples within 0.45 ± 0.001 were selected to test negative results.

**
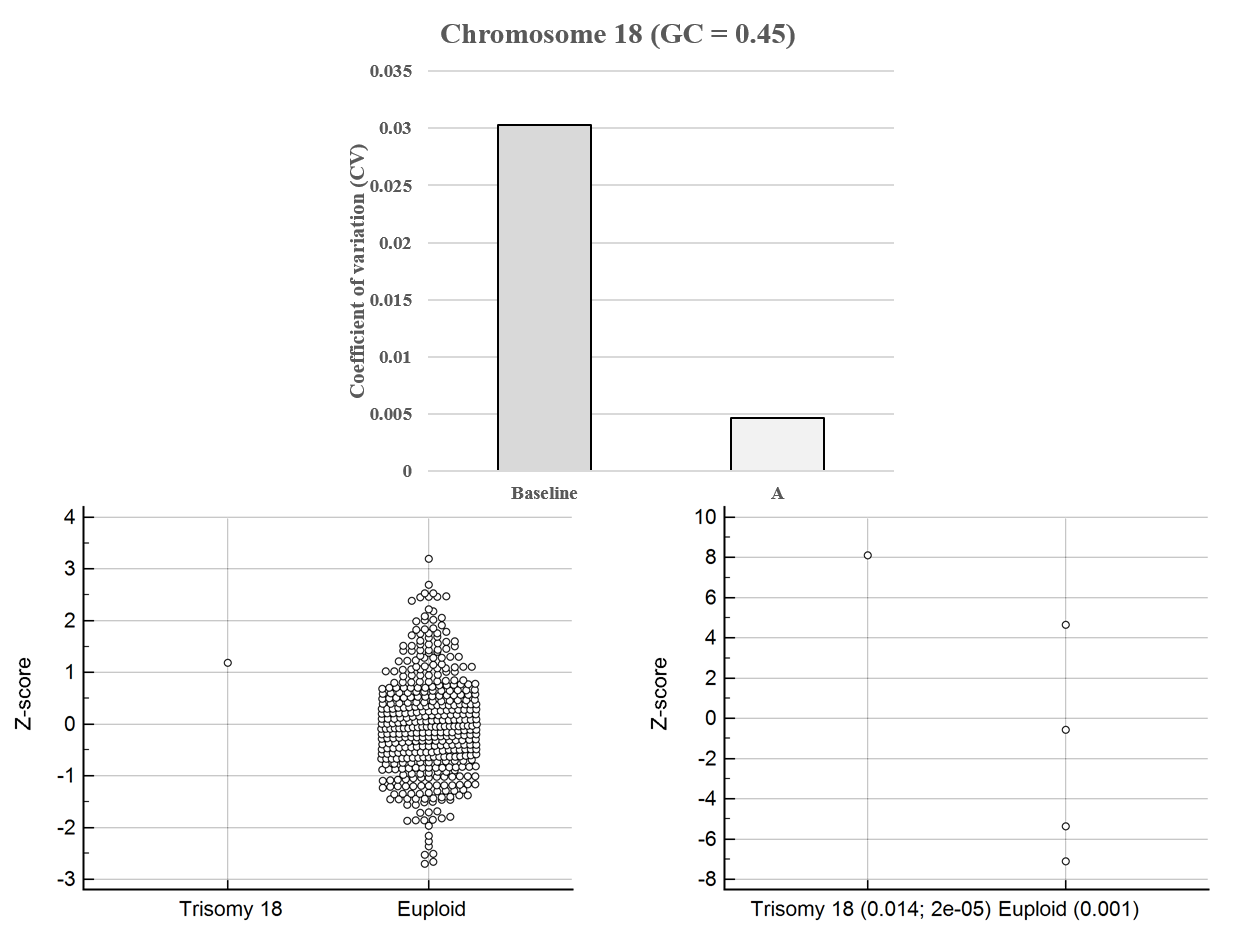
**

**Figure S6 – Coefficient of variation (CV) and z scores for chromosome 13 with and without the adaptive method using the only sample with a GC = 0.421**

The baseline bar represents the coefficient of variation used to measure the genomic representation of chromosome 18 among reference samples (*n* = 396) without adaptive sample selection. The additional bar represents the CV with adaptive sample selection. The bar marked A represents the coefficient of variation used to measure the genomic representation of chromosome 13 among selected reference samples (*n* = 177). We had only one T13 sample and could not test the results using the other T13 samples. However, we found that only the T13 sample could generate a good set of adaptively selected samples (*n* = 177), which represented 0.017 and 0.0001 for a GC range and a reads fraction range of the test sample, respectively, in the set of adaptive reference samples. Z scores obtained for positive and negative samples were separated more clearly by the unambiguous threshold using the adaptive method for chromosome 13. The euploid samples within 0.421 ± 0.001 were selected to test negative results.


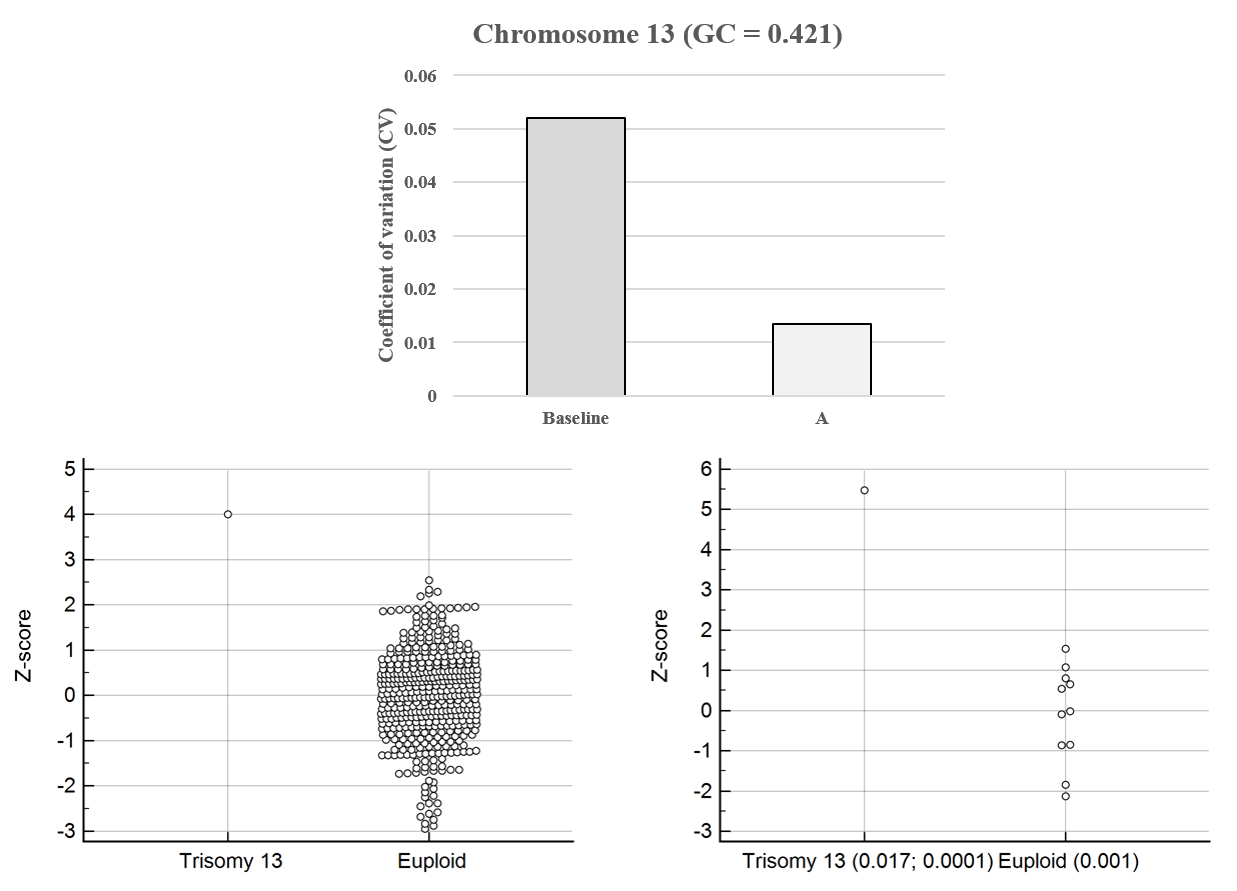


**Figure S7 – Relationship of the reads fractions and the GC contents of samples fitted to a linear model**

All chromosomes are represented. Black dots represent reads fractions vs. the GC contents of samples among the euploid controls (*n* = 396) as confirmed by karyotyping.

**
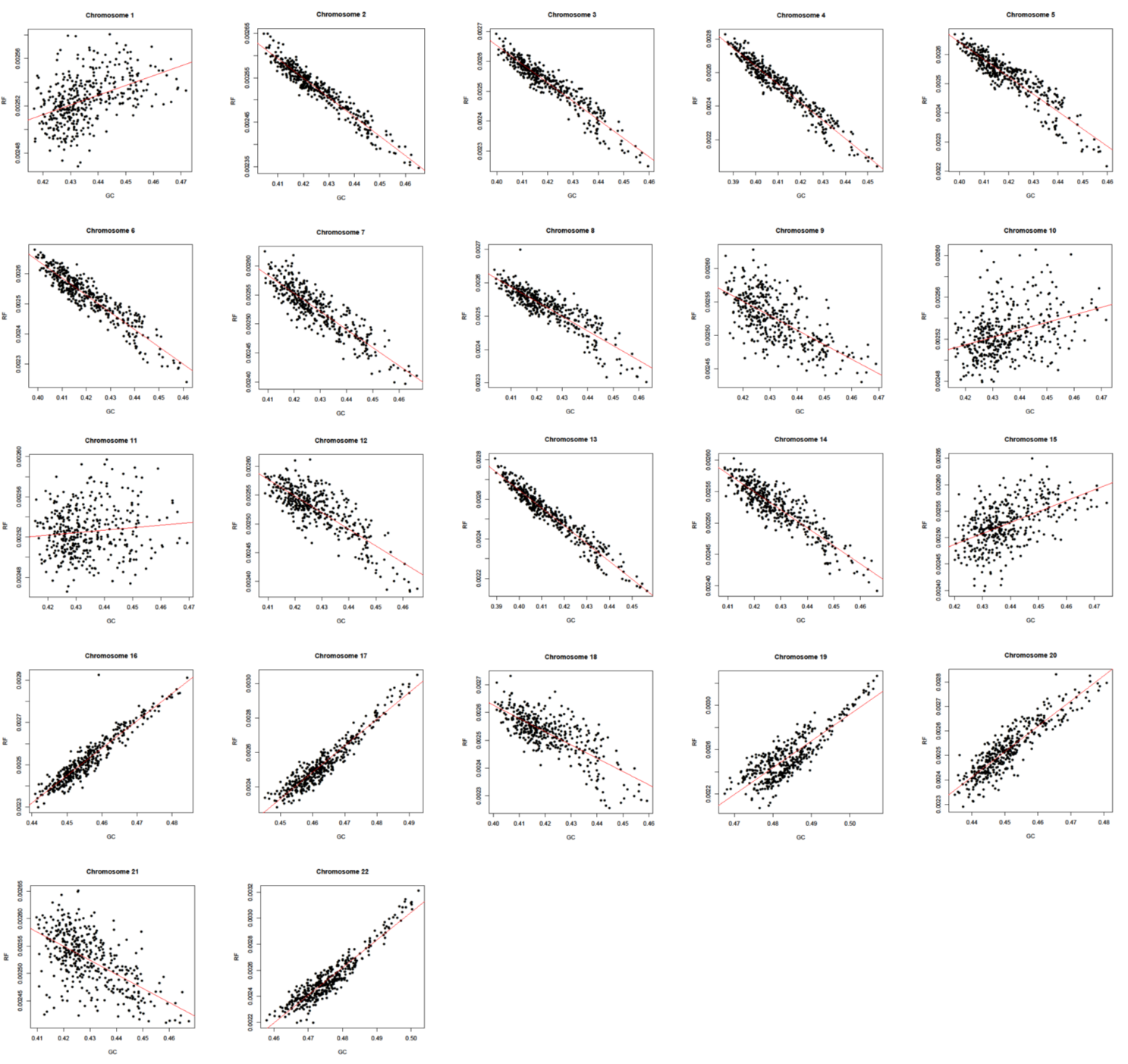
**
